# 

**Published:** 1899-08

**Authors:** 


					﻿THE DENTAL REGISTER,
A. Monthly Magazine Devoted to the Interests of the
DENTAL PROFESSION.
Terms Reduced to $2.00 Per Year. Single Copies, 25 Cents.
EDITED BY PROF. J. TAFT, D.D.S,
------AND------
Published by SAM'L A. CROCKER & CO., Ohio Dental and Surgical Depot,
The volume commences in January and ¿loses in December.
The Register being issued monthly is always fresh, therefore Indispensable
to the practitioner who desires to keep posted up with the new inventions and
improvements which are daily developed. Neither expense nor effort will be
spared to give value and variety to its contents Articles will be illustrated with
well executed engravings whenever they will add to the interest or assist to ex-
planation.	.
Its extensive circularon and frequency of issue make it one of the BEST DEN-
TAL ADVERTISING MEDIUMS in the United States.
All subscriptions must begin with January or July.
Local or special notices, five lines or less, will be inserted for $3 each insertion ;
¡rver five lines, 50 cts. per line.
All advertising bills payable in advance, or at furthest, after the issue of the
first number containing the advertisement. All transient advertisements to be
>aid for in advance.
««-CONTRIBUTIONS SOLICITED.
Exclusively Editorial Communications should be addressed to the Editor.
The latest number of the Register may be ordered with or without other good
any time, and all letters should be a.ddreP'~J
				

